# Topics of the Month

**Published:** 1893-06

**Authors:** 


### TOPICS OF THE MONTH.

By the way, who is M. Allen Starr, M. D., anyhow?

---

The Life Underwriters' Association, of Western New York, tendered their medical examiners of Buffalo a banquet at the Genesee, Wednesday evening, May 17, 1893. Among the physicians present were Drs. Phelps, Hopkins, Clark, Hayd, B. Long, E. Long, Ingraham, Goltra, Heath, Howell, Flagg, Preiss, and Krauss.

---

The Fresh Air Mission is a charity which ought to, and no doubt does, appeal to the sympathies of every humane person. The good work that has been done heretofore by this organization is a sufficient guarantee that whatever contributions it receives will be faithfully and economically applied. The proposition to enlarge the scope of the Mission and to erect buildings for its special needs, is one well deserving the consideration and liberal contributions of the citizens of Buffalo.

Much has been done in the past to benefit the children, but still more can be done, and ought to be done, by the philanthropic and liberal-minded citizens of Buffalo. Let the contributions pour in liberally, and the results will be speedily manifested. If there is any class especially deserving, it is the children. Whatever their physical, mental, or other defects, they are not responsible for their environment or their feebleness. Let every one remember the mandate of our Saviour, "Suffer little children to come unto me, for of such is the kingdom of heaven."

---

INASMUCH as journals of every grade, color, description, and language have had their say on the question of Sunday opening of the Columbian Exposition, so the JOURNAL may be pardoned for expressing its opinion, or rather the sentiment of the medical profession. Physicians who go to Chicago, with but very few exceptions, will have very limited time to spend and surely will wish to make the very best use of that time, and that time will be at the Exposition grounds whether it be Sunday or Friday. Our suggestion to the Columbian Commission is to throw the gates open every day in the week.

---

IN THE announcement of the Chicago Post-Graduate Course, Dr. Emery Lanphear, who resides in Kansas City, Mo., was credited with a residence in New York. Dr. Lanphear will lecture upon Some Achievements in Intracranial Surgery, and illustrate his subject from cases coming within the scope of his own personal experience and upon which he has operated, thus giving his lectures an extremely practical character, which is a hundred-fold better than theory.

Dr. Lanphear has had a remarkable experience in injuries and surgical diseases of the brain, and he has been singularly successful in their management.

---

THE New York State Reformatory, at Elmira, has recently published its seventeenth year book. It includes the annual report of the Board of Managers for the year ending September 30, 1892.

We wish this book could be in the hands of every philanthropist, educator, and all interested in the subject of prison reform. In this volume is told the methods which are successfully employed to recover young criminals, or unfortunate lads, from the road to destruction, and the story of their reform is an interesting one.

The purpose of the Elmira Reformatory is to take charge of first criminal offenders of ages from sixteen to thirty. This embraces the period of mental plasticity during which they are presumed to be reclaimable. It is during this period that they can be, if at all, returned to society as citizens and self-supporting workers. We have seldom seen so interesting a record of educational, physical, and moral training as is recorded in this volume. It is handsomely printed on heavy book paper, profusely illustrated, and neatly bound. We are indebted to Dr. William C. Wey for a copy.

Dr. John Edwin Rodes, of Chicago, has compiled a very useful little pocket dose book. It is quite compact and adapted to the vest pocket. It contains a complete posological table of old remedies, and such new ones as are of value. The doses are given in both apothecaries' weights and measures, and in the metric system, with an easy rule for changing from the one to the other. It also contains rules for the practice of artificial respiration, the treatment of asphyxia, a table of poisons and their antidotes, together with many other useful information. It is published by Sharpe & Smith, Chicago, to whom orders should be addressed.

The question of good roads is one that requires considerable agitation. It is not only in the interest of agriculture and social interchange of courtesies between the inhabitants of a county and state, but it is also in the interest of good health that this reform should be urged. Especially is it of necessity that the agricultural interests of the country should be roused on this subject, because in the success of the farmer lies the prosperity of the commonwealth. Col. Albert A. Pope, of Boston, has worked indefatigably in this direction, and deserves much credit for his generous and untiring efforts. It is desirable that the Senate Committee on Agriculture in the Fifty-third Congress, of which the Hon. James J. George, of Carrollton, Miss., is Chairman, should be urged to investigate the relation of roads to the agricultural interests of the country. To this end we hope every interested person will address Mr. George either by letter or petition. There is no half-way point in this matter; we must either have good roads or else let them go by default in the future as they have in the past.

